# Factors Associated With the Autonomy of Patients Treated by Haemodialysis in Community Centres. A Cross‐Sectional Study

**DOI:** 10.1111/jorc.70088

**Published:** 2026-10-22

**Authors:** Akram Achour, Clémence Béchade, Thierry Lobbedez, Stéphane Edet, Maxence Ficheux, Sonia Guillouët

**Affiliations:** ^1^ Santelys Association Loos France; ^2^ Department of Health Training and Research Caen University Caen France; ^3^ Nephrology Department Caen University Hospital Caen France; ^4^ Research Unit INSERM U1086, Anticipe Caen France; ^5^ ANIDER Association Rouen France

**Keywords:** autonomy, haemodialysis, nurses, satellite unit, self‐care unit

## Abstract

**Background:**

In France, haemodialysis is delivered in different settings depending on patients' health status and autonomy. Community centres include self‐care and satellite units, which vary in organisation and patient involvement.

**Objectives:**

To describe the characteristics of patients treated in haemodialysis community centres and to identify factors associated with autonomy in self‐care units.

**Design:**

Cross‐sectional study.

**Participants:**

All patients receiving haemodialysis in community centres in a French region on December 31, 2021.

**Measurements:**

Patient characteristics and multiple dimensions of patient autonomy were recorded. Multivariate analyses assessed associations between patient factors and technical autonomy.

**Results:**

Among 507 patients, 304 were treated in self‐care units and 203 in satellite haemodialysis units. Patients in satellite haemodialysis units had a median age of 69 years (IQR 59–78; range 24–90), compared with 66 years (IQR 54–75; range 22–91) in self‐care haemodialysis units. They also had more comorbidities. Younger age was associated with greater autonomy in arteriovenous fistula puncture‐site dressing (PR 0.97, 95% CI 0.96–0.98), setting the haemodialysis machine (PR 0.97, 95% CI 0.96–0.98), and programming it (PR 0.97, 95% CI 0.95–0.98). Autonomy differed significantly between centres, with centre affiliation significantly associated (*p* < .001) with autonomy for the arteriovenous fistula puncture‐site dressing and machine‐related tasks.

**Conclusions:**

Patient characteristics in self‐care haemodialysis units did not fully explain autonomy for haemodialysis tasks. Considerable variability between centres was observed, suggesting that centre‐related factors may contribute to differences in patient autonomy.

## 1 Introduction

Approximately 700 million people are estimated to have CKD worldwide (Francis et al. 2024). Among kidney replacement therapies, haemodialysis (HD) is the most widely used worldwide (Bello et al. 2024). In France, 53.359 patients with kidney failure were receiving dialysis in 2024, of whom 95% were treated by haemodialysis (‘French Report REIN’ 2024). In the French health care system, in‐centre dialysis facilities must be located inside a hospital with an intensive care unit (Decree No. 2002‐1198, 2002‐1198, 2002). Patients receiving haemodialysis can also be treated in self‐care dialysis centres (SCHD) or satellite dialysis centres (SHDU), where there is no nephrologist on site, as opposed to in‐centre dialysis units. Regional discrepancies exist in the use of self‐care haemodialysis in France, with the proportion of patients ranging from less than 1% to approximately 30% across regions in 2024; the proportion was 14.1% in the Normandy Region and 13.5% nationally. In principle, SCHD and SHDU differ in the expected level of patient autonomy and nursing support, with greater patient participation in dialysis‐related tasks expected in SCHD than in SHDU. The choice between SCHD and SHDU is based on comorbidity, cardiovascular tolerance of the dialysis session, and the level of patient autonomy (Canaud and Couchoud 2022). The mandatory nurse‐patient ratio ranges from 1:8 to 1:6 in SCHD, depending on the level of assistance required, compared with 1:4 in SHDU. Dialysis nurses can assist patients who are not completely autonomous with various tasks, such as arteriovenous fistula (AVF) puncture.

To be treated in a SCHD, patients must first receive specific training from dialysis nurses. During this training, patients learn to perform and monitor dialysis‐related tasks, including weighing, blood pressure monitoring, preparation of the dialysis machine, connection and disconnection of the extracorporeal circuit, and monitoring of the dialysis session. Once the patient is considered autonomous for treatment, a transfer is organized from the in‐centre dialysis facility to the SCHD (Loos‐Ayav et al. 2008).

## 2 Literature Review

Self‐care in dialysis extends beyond adherence to treatment and includes behaviours related to disease and treatment management, as well as activities aimed at promoting personal well‐being and adaptation to living with chronic disease (Ricka et al. 2002). In haemodialysis, self‐care may involve patient participation in dialysis‐related tasks. Greater participation in these tasks has been associated with higher levels of patient activation and a positive experience of involvement in care (Wilkie and Barnes 2019). Patient education is also an important component of self‐management in haemodialysis (van Eck van der Sluijs et al. 2021). Training nurses to support patient involvement in haemodialysis‐related tasks has been implemented in shared haemodialysis care programmes (Barnes et al. 2013). More recently, a qualitative study identified organizational and staff‐related factors that may influence patients' involvement in haemodialysis treatment tasks (Kurtz et al. 2026).

However, patient participation in haemodialysis care remains variable. In the study by Kurtz et al. (2025), participation was defined as engagement in at least two care tasks, regardless of the extent of involvement. Using this criterion, about 40% of patients were considered as participating, but all were involved only in simple care tasks. None of the patients engaged in the seven care tasks classified as complex. As the study included both hospital‐based and community‐based (private) facilities, its findings may not reflect the level of autonomy of patients treated in SCHD or SHDU.

Although patient autonomy is central to the organization of these dialysis settings, the level of autonomy achieved by patients treated in SCHD and SHDU has been poorly documented. The aim of our study was therefore to assess patients' autonomy in SCHD and SHDU and to determine which patient characteristics were associated with autonomy.

## 3 Materials and Methods

### 3.1 Study Design

This cross‐sectional study was conducted to assess the autonomy dimensions of patients treated in SCHD and SHDU in a French region. Patient characteristics were extracted from the database of the Renal Epidemiology and Information Network (REIN) and from medical charts. Age at HD initiation, sex, and the following comorbidities were obtained from the registry: diabetes mellitus, cause of kidney disease, cardiovascular and neurological comorbidities. Patient comorbidities were assessed using the modified Charlson Comorbidity Index adapted for patients with kidney failure, with higher scores indicating a greater comorbidity burden (Hemmelgarn et al. 2003). Measured patient autonomy was obtained from routine clinical assessments, whereas perceived autonomy was collected using a questionnaire.

This study was reported in accordance with the Strengthening the Reporting of Observational Studies in Epidemiology (STROBE) guidelines (von Elm et al. 2007).

### 3.2 Study Population

Patients treated by haemodialysis for more than 3 months in the 27 SCHD units and the 6 SHDU units of the Normandy Region, on December 31, 2021, were included in the study. All these units are managed by the same dialysis association. Patients were informed about the study and their right to object to the use of their data for research purposes. An additional questionnaire was proposed to patients treated in the SCHD units, and participation was voluntary.

### 3.3 Assessment of Autonomy

Patient autonomy in haemodialysis should not be limited to the performance of technical tasks. Årestedt et al. (2019) showed that patient participation in dialysis care encompasses several aspects, including sharing information and knowledge, learning, involvement in decision‐making, treatment management and self‐care activities. In this study, four dimensions of patient autonomy were considered: technical, clinical, functional, and organizational autonomy. Technical autonomy refers to the patient's ability to perform the technical tasks required for self‐care dialysis. Clinical autonomy refers to the degree to which the patient is able to control the clinical decisions associated with his or her health situation (Ladin et al. 2018; Peng et al. 2019). Organizational autonomy refers to patients' independence from health care institutions to carry out haemodialysis tasks (McLaughlin et al. 2003), while functional autonomy is the ability to perform activities of daily living independently (Struhkamp 2005).

### 3.4 Data Collection

In order to assess the care needs of patients receiving dialysis, nurses and nursing assistants from the home dialysis association ANIDER collect patient data as part of routine clinical care. Data collection follows a standardized procedure across all participating sites, using a common data collection form. These assessments were part of routine clinical practice before the study. Some of these data, reflecting patient autonomy, were used for the study. Only data relating to the four dimensions of autonomy mentioned above were extracted, including the following tasks and abilities: moving around, dressing and undressing, eating, administering treatment, measuring blood pressure, weighing oneself, inserting the needle into the puncture site; but also, hearing and seeing correctly, being continent, and communicating easily.

Measured autonomy was based on assessments routinely collected by nurses and nursing assistants to evaluate patients' care needs, whereas perceived autonomy was based on patients' self‐assessment. Then, to complement the assessment performed by HD teams, a four‐item binary questionnaire was administered to consenting patients treated in the SCHD units. The questionnaire was limited to SCHD patients because this dialysis setting is intended for patients with a greater level of autonomy and involvement in their dialysis care. It was developed specifically for this study and was not a validated instrument. It had not been formally piloted prior to the study and was administered in paper format. For example, one item assessed whether patients were able to identify mild complications related to dialysis (yes/no).

Measures included in each autonomy dimension and their classification as measured or perceived are summarized in Table 1.

**Table 1:** Measures used to assess the four dimensions of patient autonomy.

Autonomy dimensions	Measured autonomy (caregiver assessment)	Perceived autonomy (patient self‐assessment)^a^
Clinical autonomy	—	Good knowledge of serious complications
		Able to identify mild complications
		Able to solve complications without help
Functional autonomy	Mobilization	—
	Dress and undress	
	Vision, hearing, continence	
	Eating	
	Communication	
Organizational autonomy	Blood pressure measurement	Perceived autonomy in dialysisrelated tasks
	Treatment administration	
	Needle insertion	
	Weight	
Technical autonomy	Programming HD machine	—
	Setting up HD machine	
	Puncture‐site compression	
	AVF puncture‐site dressing	

a With a four‐item binary questionnaire.

### 3.5 Statistical Analysis

Frequencies and percentages were used to describe categorical variables, while continuous variables were expressed as medians and interquartile ranges (IQR).

Factors associated with technical autonomy in SCHD were investigated using bivariate and multivariate Cox regression models with robust variance. Four separate models were performed, corresponding to the four binary measures of technical autonomy: HD machine programming, HD machine setting up, puncture‐site compression and puncture‐site dressing. Variables with a p value lower than 0.20 in the bivariate analysis were included in the multivariate models. As the rate of the event of interest was greater than 10%, the odds ratio (OR) could overestimate the prevalence ratio (PR); therefore Cox regression with robust variance, assigning the same arbitrary time value to all participants, was used to estimate prevalence ratios (Barros and Hirakata 2003).

The interactions between diabetes, modified CCI, and cardiovascular diseases were tested. Statistical analyses were performed with R 4.1.1 (R Foundation for Statistical Computing).

### 3.6 Missing Data

The rate of missing data was less than 1% for each covariate. Nevertheless, multiple imputation by chained equations was performed for all missing data.

### 3.7 Statement of Ethics

This study was conducted in accordance with the Declaration of Helsinki (World Medical Association 2025) and informed consent was obtained from all participants. In compliance with French law, patients were informed and had the opportunity to express their opposition. Written informed consent was obtained from participants who completed the additional questionnaire.

Data were obtained from the French Renal Epidemiology and Information Network (REIN) through the regional coordination unit. The database was anonymized before analysis, and the investigator had no access to patient‐identifying information.

The protocol was reviewed and approved by the Research and Ethics Committee of Caen University Hospital, 4th October 2021, with the approval number ID 6526.

## 4 Results

### 4.1 Descriptive Study

#### 4.1.1 Patient Characteristics

A total of 507 patients treated with HD in 27 SCHD and 6 SHDU were included. Those individuals had been trained by the nephrology teams of 13 in‐centre dialysis units. Of the 507 patients, 304 and 203 were treated in a SCHD and in a SHDU, respectively. Patients treated in satellite haemodialysis units had a median age of 69 years (IQR 59–78; range 24–90), compared with 66 years (IQR 54–75; range 22–91) in self‐care haemodialysis units. The median modified CCI of the populations included was 2 (IQR: 0–4) and was higher in the group of patients treated in SHDU than in the group of patients treated in SCHD (3 (IQR: 0–5) vs. 2 (IQR: 0–3,5)). The sex ratio (M/F) was 2.07, and 187 (36.9%) patients had diabetes. A total of 133 (26.2%) patients included in this study were registered on the transplantation waiting list. Patient characteristics are presented in Table 2.

**Table 2:** Patient characteristics.

	General population		Self‐care unit (SCHD)		Satellite unit (SHDU)	
Covariates	*N* = 507 Median (IQR)		*N* = 304 Median (IQR)		*N* = 203 Median (IQR)	
Age per years	67 (57–76) Range 22–91		66 (54–75) Range 22–91		69 (59–78) Range 24–90	
CCI	4 (2‐5)		3 (2‐5)		4 (2‐6)	
Modified CCI	2 (0‐4)		2 (0‐3)		3 (0‐5)	
Time spent in dialysis per months	47 (22‐90)		46 (21‐87)		49 (22‐91)	

	***N***	***%***	***N***	***%***	***N***	***%***
Age per tertiles						
22–61 years	165	32.5	110	36.2	55	27.1
62–73 years	170	33.5	100	32.9	70	34.5
> 73 years	172	33.9	94	30.9	78	38.4
Sex (male)	342	67.5	213	70.1	129	63.5
Modified CCI by category						
0	178	35.1	120	39.5	58	28.6
1	30	5.9	16	5.3	14	6.9
2	72	14.2	47	15.5	25	12.3
3	75	14.8	45	14.8	30	14.8
4	47	9.3	22	7.2	25	12.3
5	37	7.3	19	6.3	18	8.9
≥ 6	68	13.4	35	11.5	33	16.3
Cardiovascular disease	151	29.8	79	26	72	35.5
* Missing data*	16	—	14	—	4	—
Diabetes	187	36.9	106	34.9	81	39.9
* Missing data*	3	—	1	—	2	—
Cause of kidney disease						
Diabetic kidney disease	87	17.2	50	16.4	37	18.2
Glomerular disease	95	18.7	60	19.7	35	17.2
Tubulointerstitial disease	35	6.9	16	5.3	19	9.4
Polycystic kidney disease	44	8.7	31	10.2	13	6.4
Vascular kidney disease	72	14.2	42	13.8	30	14.8
Other	88	17.4	56	18.4	32	15.8
Unknown	86	17.0	49	16.1	37	18.2
Inscription on the transplantation waiting list	133	26.2	76	25	57	28.1
Distance between the patient's HD unit and in‐center HD						
[1, 10] km	383	75.54	180	59.2	203	100
[10, 30] km	40	7.89	40	13.2	0	0
[30, 60] km	84	16.57	84	27.6	0	0

Abbreviations: CCI, Charlson Comorbity Index; HD, haemodialysis; IQR, interquartile range.

### 4.2 Measured Level of Autonomy

#### 4.2.1 Functional Autonomy

Patients treated in SCHD were more frequently able to mobilize independently than those treated in SHDU (96.4% vs. 90.6%) and to dress and undress independently (95.7% vs. 91.6%).

#### 4.2.2 Technical Autonomy

In SCHD, 51.6% (*n* = 157/304) were able to set up HD machines alone, 34.9% (*n* = 106/304) were able to program the dialysis treatment, 87% (*n* = 262/304) were able to achieve puncture‐site compression, and 40.7% (*n *= 123/304) were able to dress puncture site.

The HD machine was set up independently by 2.5% (*n* = 5/203) of patients, and programmed independently by 3.9% (*n* = 8/203). 58.6% (*n *= 119/203) of them compressed puncture site and 11.4% (*n* = 23/203) achieved puncture‐site dressing.

Variability in patients' technical autonomy level according to the SCHD themselves is shown in Figure 1 and in SCHD according to the in‐centre HD unit in which the patient was trained in Figure 2.

**Figure 1:**
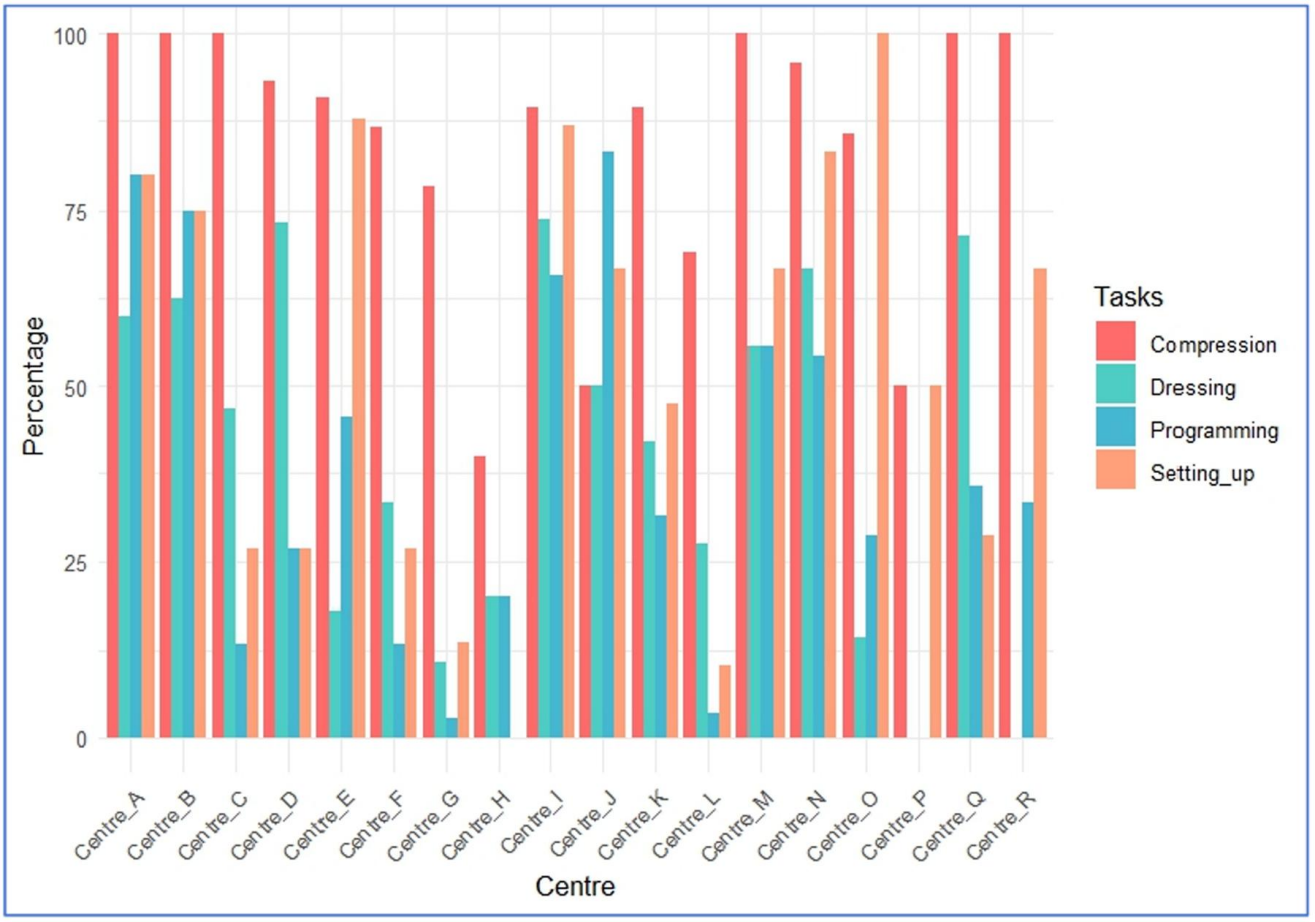
Variability in patients' technical autonomy level according to the self‐care units.

**Figure 2:**
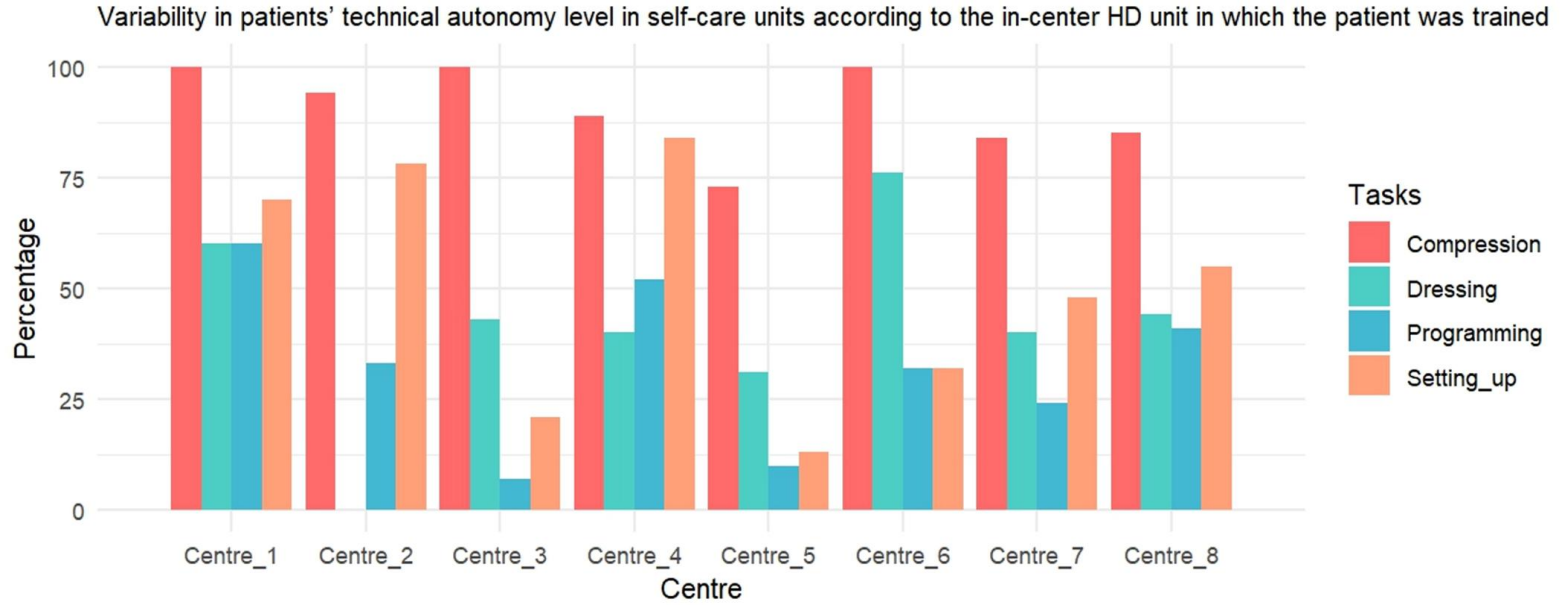
Variability in patients' technical autonomy level in self‐care units according to the in‐center HD unit in which the patient was trained.

#### 4.2.3 Organizational Autonomy

In both types of units, most patients were able to weigh themselves (95.7% in SCHD and 90.6% in SHDU). Blood pressure was measured by 49.8% of patients in SCHD and 17.2% in SHDU. Needle insertion was performed by 2% of patients in both SCHD and SHDU. Complete data on measured autonomy are presented in Table 3.

**Table 3:** Measured autonomy (data from each patient recorder by nurses).

Covariates	General population		Self‐care unit (SCHD)		Satellite unit (SHDU)	
	*N*	*%*	*N*	*%*	*N*	*%*
*Functional Autonomy*						
Mobilization						
Performed by the patient	477	94.1	293	96.4	184	90.6
Required assistance from the nurse	30	5.9	11	3.6	19	9.4
Dress and undress						
Performed by the patient	476	94.1	291	95.7	185	91.6
Required assistance from the nurse	30	5.9	13	4.3	17	8.4
Missing data	1	—	0	0	1	—
Vision, hearing and/or continence						
Non deficient	354	70	213	70.3	141	69.5
Deficient	152	30	90	29.7	62	30.5
Missing data	1	—	1	—	0	0
Eating						
Performed by the patient	503	99.6	301	99.7	202	99.5
Required assistance from the nurse	2	0.4	1	0.3	1	0.5
Missing data	2	—	2	—	0	0
Communication						
Able to speak clearly	480	94.7	291	95.7	189	93.1
Able to have consistent statements	473	93.3	284	93.4	189	93.1
*Technical Autonomy*						
HD machine setting up						
Performed by the patient	162	32	157	51.6	5	2.5
Required assistance from the nurse	344	68	147	48.4	197	97.5
Missing data	1	—	0	—	1	—
HD machine programming						
Performed by the patient	114	22.5	106	34.9	8	3.9
Required assistance from the nurse	393	77.5	198	65.1	195	96.1
Puncture‐site compression						
Performed by the patient	381	75.6	262	87	119	58.6
Required assistance from the nurse	123	24.4	39	13	84	41.4
Missing data	3	—	3	—	0	—
AVF puncture‐site dressing						
Performed by the patient	146	29	123	40.7	23	11.4
Required assistance from the nurse	358	71	179	59.3	179	88.6
Missing data	3	—	2	—	1	—
Optimal technical autonomy						
Patient is able to perform all the tasks	59	11.64	58	19.08	1	0.5
*Organizational Autonomy*						
Blood pressure measurement						
Performed by the patient	186	36.8	151	49.8	35	17.2
Required assistance from the nurse	320	63.2	152	50.2	168	82.8
Missing data	1	—	1	—	0	0
Treatment administration						
Performed by the patient	381	75.3	231	76.2	150	73.9
Required assistance from the nurse	125	24.7	72	23.8	53	26.1
Missing data	1	—	1	—	0	0
Needle insertion						
Performed by the patient	10	2	6	2	4	2
Required assistance from the nurse	497	98	298	98	199	98
Weight						
Performed by the patient	472	93.65	289	95.7	183	90.6
Required assistance from the nurse	32	6.35	13	4.3	19	9.4
Missing data	3	—	2	—	1	—

### 4.3 Perceived Level of Autonomy

#### 4.3.1 Clinical Autonomy

Of the 304 patients invited to complete the questionnaire, 144 responded, corresponding to a response rate of 47%. Among them, 77.5% (*n* = 100) were able to identify serious complications. However, 28.9% (*n* = 37) of patients could resolve complications without help.

#### 4.3.2 Organizational Autonomy

Overall, 60.6% (*n* = 77) of respondents perceived themselves as autonomous in dialysis‐related tasks. Data are presented in Table 4.

**Table 4:** Patient's perceived autonomy level (added questionnaire) in the self‐care units.

Covariates	Self‐care unit (SCHD) *N* = 144/304	
	*N*	*%*
*Clinical Perceived Autonomy*		
Good knowledge of serious complications		
Yes	100	77.5
No	29	22.5
Missing data	15	—
Able to identify mild complications		
Yes	108	83.1
No	22	16.9
Missing data	14	—
Able to solve complications without help		
Yes	37	28.9
No	91	71.1
Missing data	16	—
*Organizational Perceived Autonomy*		
Perceived autonomy related to dialysis' tasks		
Yes	77	60.6
No	50	39.4
Missing data	17	—

*Note:* Percentages were calculated based on available data.

### 4.4 Factors Associated With Autonomy in Self‐Care Haemodialysis Units

In the bivariable analysis, age and in‐centre HD facility in which patients were trained before transfer to SCHD were significantly associated with the technical autonomy level across all four models. Diabetes was significantly associated with puncture‐site compression and HD machine programming, cardiovascular disease with HD machine setup and programming, and sex with HD machine setup.

In the multivariable analysis, no factors were significantly associated with puncture‐site compression. Age and the in‐centre dialysis facility in which patients were trained were significantly associated with autonomy in puncture‐site dressing (age: PR 0.97, 95% CI 0.96–0.98, *p* < 0.001; facility: global *p* < 0.001), HD machine setup (age: PR 0.97, 95% CI 0.96–0.98, *p* < 0.001; facility: global *p* < 0.001), and HD machine programming (age: PR 0.97, 95% CI 0.96–0.99, *p* < 0.001; facility: global *p* < 0.001). The results of the multivariate analysis are presented in Table 5.

**Table 5:** Analysis of factors associated with patient autonomy in self‐care units (SCHD) bivariate analysis.

Covariates	Puncture‐site compression Model			AVF puncture‐site dressing Model			HD machine setting Model			HD machine programming Model		
	PR	CI 95%	*p* value	PR	CI 95%	*p* value	PR	CI 95%	*p* value	PR	CI 95%	*p* value
**Bivariate analysis**												
Age	0.99	(0.98–1.00)	0.03	0.97	(0.96–0.98)	< 0.001	0.97	(0.96–0.98)	< 0.001	0.97	(0.95–0.98)	< 0.001
Modified CCI	0.97	(0.94–1.03)	0.55	0.98	(0.92–1.04)	0.49	0.98	(0.92–1.03)	0.42	0.99	(0.92–1.07)	0.87
Cardiovascular disparities	0.92	(0.70–1.22)	0.55	1.08	(0.72–1.61)	0.70	0.72	(0.49–1.05)	0.08	0.70	(0.43–1.13)	0.13
Diabetes	0.75	(0.58–0.98)	0.03	0.82	(0.56–1.21)	0.32	0.92	(0.66–1.28)	0.62	0.72	(0.47–1.11)	0.13
Sex (male)	1.20	(0.86–1.53)	0.27	0.85	(0.58–1.24)	0.40	1.37	(0.95–1.98)	0.09	0.99	(0.64–1.51)	0.95
*In‐center HD unit*												
Center 1	Ref		0.08	Ref		< 0,001	Ref		< 0.001	Ref		< 0.001
Center 2	0.91	(0.41–2.00)		0.00	0.00 Inf		1.20	(0.49–2.98)		0.46	(0.15–1.42)	
Center 3	1.12	(0.49–2.54)		0.62	(0.20–1.94)		0.21	(0.05–0.80)		0.08	(0.01–0.69)	
Center 4	0.84	(0.42–1.67)		0.57	(0.23–1.38)		1.40	(0.63–3.08)		0.82	(0.34–1.96)	
Center 5	0.57	(0.28–1.17)		0.41	(0.16–1.06)		0.12	(0.04–0.35)		0.11	(0.03–0.37)	
Center 6	1.15	(0.54–2.42)		1.46	(0.58–3.65)		0.33	(0.12–0.91)		0.43	(0.15–1.25)	
Center 7	0.76	(0.35–1.65)		0.57	(0.21–1.57)		0.56	(0.22–1.43)		0.31	(0.10–0.96)	
Center 8	0.77	(0.39–1.51)		0.65	(0.28–1.54)		0.67	(0.30–1.49)		0.59	(0.25–1.40)	
**Multivariate analysis**												
Age	0.99	(0.98–1.00)	0.10	0.97	(0.96–0.98)	< 0.001	0.97	(0.96–0.98)	< 0.001	0.97	(0.96–0.99)	< 0.001
Modified CCI	—	—		—	—		—	—		—	—	
Cardiovascular disparities	—	—		—	—		0.95	(0.63–1.45)	0.82	0.92	(0.55–1.53)	0.74
Diabetes	0.84	(0.63–1.10)	0.20	—	—		—	—		0.81	(0.51–1.28)	0.37
Sex (male)	—	—					1.44	(0.98–2.11)	0.6	—	—	
In‐center HD unit												
Center 1	Ref		0.21	Ref		< 0.001	Ref		< 0.001	Ref		< 0.001
Center 2	0.91	(0.41–1.00)		NA	NA		1.58	(0.63–3.93)		0.64	(0.20–2.00)	
Center 3	1.12	(0.49–2.59)		0.89	(0.28–2.77)		0.28	(0.07–1.10)		0.13	(0.01–1.06)	
Center 4	0.84	(0.42–1.67)		0.73	(0.30–1.80)		1.83	(0.83‐4.05)		1.14	(0.47–2.77)	
Center 5	0.57	(0.28–1.17)		0.61	(0.24‐1.59)		0.18	(0.06‐0.52)		0.18	(0.05–0.61)	
Center 6	1.15	(0.54–2.42)		2.29	(0.90‐5.86)		0.51	(0.18‐1.43)		0.75	(0.25–2.23)	
Center 7	0.76	(0.35–1.65)		0.79	(0.28‐2.19)		0.93	(0.36‐2.43)		0.49	(0.15–1.56)	
Center 8	0.77	(0.39–1.51)		0.95	(0.40‐2.27)		1.00	(0.45‐2.26)		0.94	(0.39‐2.27)	

Abbreviations: AVF, arteriovenous fistula; CI, confidence interval; CCI, Charlson Comorbity Index; HD, haemodialysis; PR, prevalence ratio.

## 5 Discussion

Age was the main patient characteristic associated with technical autonomy in our study, whereas comorbidities did not explain the level of autonomy. Age has previously been identified as a factor to consider when aiming to improve self‐care management and clinical outcomes in haemodialysis patients (Kim et al. 2019).

Evaluation of the physical functioning of patients receiving haemodialysis is recommended by the European Renal Best Practice guidelines (Farrington et al. 2016), as decreased physical functioning negatively affects quality of daily life and autonomy in this population (Altintepe et al. 2006; Matsuzawa et al. 2014). In this study, more than 90% of patients in both SCHD and SHDU were independent in mobilization, dressing and undressing, eating, and communication, although 30% had a deficit in vision, hearing and/or continence. These findings are consistent with a recent French pilot study conducted by Huré et al. (Huré et al. 2025), which assessed the impact of dialysis‐related autonomy on patients' occupational status, physical activity, social engagement, and quality of life. Among 182 patients undergoing autonomous dialysis modalities, including SCHD, daily home HD, and long nocturnal home HD, 33% were actively employed, approximately 40% reported meeting recommended physical activity thresholds, and over half reported no limitations in mobility or daily activities. These results are also consistent with those of a Swedish study (Ageborg et al. 2005) that aimed to investigate potential differences in the way patients receiving haemodialysis view their quality of life and self‐care ability if they dialyze themselves at home, dialyze themselves in a SCHD, or in an outpatient dialysis unit. A similar pattern of results was obtained in a Brazilian study in which patients reported more often performing daily life tasks, including eating, moving, and getting dressed without help (Gutiérrez‐Peredo et al. 2020).

In chronic disease, patient engagement and empowerment are ways to reduce mortality and complications and to enhance clinical autonomy (Riegel et al. 2019). Self‐management is defined as the ability of an individual to manage their symptoms, lifestyle changes and physical and psychosocial consequences of health conditions, particularly for chronic diseases (Barlow et al. 2002). Patient knowledge and self‐efficacy are major factors influencing self‐management (Li et al. 2014). In our analysis, only half of the patients treated in SCHD completed the additional questionnaire (n = 144/304). Among respondents, 77% reported having good knowledge, 83% could identify minor complications, and two‐thirds felt autonomous in dialysis tasks, although many still needed assistance with problem‐solving. Several studies have shown that high perceived self‐efficacy is significantly associated with better quality of life. Studies conducted in Asian populations have identified self‐care and self‐efficacy as predictors of a better quality of life, independently of age (Tsay and Healstead 2002; Nguyen et al. 2022).

However, we observed considerable variability in patient autonomy for different HD tasks across in‐centre dialysis units. This variability may reflect differences in organisational characteristics and practices that were not measured in our study. Centre‐related variability has previously been reported for different aspects of haemodialysis care (Fink et al. 2002; Ng et al. 2021). More specifically, in the United Kingdom, the proportion of patients offered shared haemodialysis care varied considerably between centres (Breen et al. 2026). A qualitative study also identified organizational and staff‐related factors, including local practices, leadership, and staff attitudes, as factors that may influence patient participation in haemodialysis‐related tasks (Kurtz et al. 2026).

Promoting patient autonomy is central to nursing care. The concept of patient activation—encouraging individuals to take an active role in their care—helps reduce passivity and has been shown to vary with comorbidities, often being lower in patients with chronic kidney disease or diabetes (Greene et al. 2015). Empowerment initiatives, such as shared care programs, reinforce this process by enabling patients to progressively assume dialysis‐related tasks and by enhancing their overall experience (Wilkie and Barnes 2019). In these shared care experiences, nurses observed that patients valued their participation and adapted quickly to performing dialysis‐related tasks, contributing to a positive care experience. Educational interventions, predominantly delivered by nurses, have been associated with improvements in vascular access self‐management behaviours, although the available evidence remains limited (Wembenyui et al. 2025). Nurses play a key role in HD, by supporting patient independence. Patients undergoing haemodialysis are interested in, and perceive themselves capable of, participating in care tasks, although nurses often underestimate this (Kurtz et al. 2021).

To enhance autonomy, nurses should identify and build on patients' existing abilities, gradually reducing reliance on staff. Individualized strategies may improve patients' self‐management and quality of life. Recognizing barriers and facilitators allows nurses to implement tailored educational and support strategies to empower patients treated by haemodialysis and optimize outcomes (Escudero‐Lopez et al. 2024).

Importantly, all patients, including older and frail individuals, should be asked about their preferred level of involvement in care (Piccoli et al. 2017). This is particularly relevant as older patients may be less likely to be offered shared haemodialysis care, although acceptance among those offered the opportunity did not differ according to age (Breen et al. 2026).

### 5.1 Limitations

Our study has several limitations. First, no global score exists for assessing overall patient autonomy. Moreover, by design, this observational study cannot establish causality. Data were collected at a single time point, providing a cross‐sectional snapshot that cannot account for potential variations in patient autonomy over time. The retrospective nature of the registry study is also associated with possible classification bias. Moreover, residual confounders related to both the dialysis team and individual patient factors were not captured in the registry. For example, the experience of nurses in HD units may be associated with patients' level of autonomy. Additionally, unmeasured patient characteristics, such as mental health conditions, including stress, anxiety, or depression, may influence patient autonomy. Despite these limitations, the findings provide new insights into factors associated with autonomy in patients treated in SCHD, and highlight the need for further investigation into team‐related determinants.

### 5.2 Implications for Clinical Practice

In clinical practice, nurses should encourage patients to participate in dialysis‐related tasks whenever they are willing and able to do so. Recent literature suggests that opportunities for participation may also depend on centre practices: the proportion of patients offered shared haemodialysis care varied considerably between dialysis centres (Breen et al. 2026). However, patients are interested in participating in their care and perceive themselves as capable of doing so, although nurses may underestimate their willingness and abilities (Kurtz et al. 2021). Shared care programmes have also shown that patients can gradually take on dialysis‐related tasks and report a positive experience of care (Wilkie and Barnes 2019). Patient involvement should nevertheless be adapted to individual abilities, preferences and motivation, as these factors may influence engagement in self‐care.

## 6 Conclusion

This study showed that characteristics and comorbidities did not fully explain patient autonomy in self‐care HD units. Autonomy was higher in SCHD than in satellite HD units, but substantial variability between centres suggests that centre‐related factors may also contribute to differences in patient autonomy. Promoting patient involvement in dialysis tasks should be a key objective of HD teams. Future research should focus on the impact of nursing interventions, patient education, and organisational factors on autonomy.

## Author Contributions

Conceptualization: Akram Achour, Clémence Béchade, Thierry Lobbedez, Stéphane Edet, Maxence Ficheux, Sonia Guillouët. Data acquisition: Clémence Béchade, Stéphane Edet. Data analysis, data interpretation and writing original draft: Akram Achour, Sonia Guillouët. Review: Clémence Béchade, Thierry Lobbedez, Stéphane Edet, Maxence Ficheux, Sonia Guillouët.

## Conflicts of Interest

The authors declare no conflicts of interest.

## Data Availability

The data that support the findings of this study are available from the corresponding author upon reasonable request.
